# Safe Percutaneous Dilatational Tracheostomy Using a Pre‐Measured Light‐Guided Endotracheal Tube Withdrawal Technique: A Case Report

**DOI:** 10.1002/rcr2.70637

**Published:** 2026-05-28

**Authors:** Yuya Aoki, Masaki Kono, Takuo Yoshida

**Affiliations:** ^1^ Division of Intensive Care, Department of Emergency and Disaster Medicine Kashiwa Hospital, the Jikei University School of Medicine Kashiwa Chiba Japan

**Keywords:** bronchoscopy, endotracheal tube, illumination guidance, patient safety, percutaneous dilatational tracheostomy

## Abstract

Percutaneous dilatational tracheostomy (PDT) is a bedside procedure for patients requiring prolonged mechanical ventilation. Although its overall safety is comparable to that of surgical tracheostomy, complications related to endotracheal tube (ETT) manipulation remain a concern. In this report, we describe a simple, reproducible two‐step approach for ETT repositioning during PDT that (1) pre‐measures an individualized withdrawal distance using bronchoscopic illumination and (2) confirms final positioning in real time by transillumination at the incision site in a patient requiring tracheostomy after prolonged mechanical ventilation. The procedure was completed within 20 min without complications. This technique may facilitate safer ETT repositioning during PDT.

## Graphical abstract

This report describes a practical adjunct to bronchoscopy‐guided percutaneous dilatational tracheostomy: a two‐step method in which an individualized endotracheal tube withdrawal distance is estimated using bronchoscopic transillumination and subsequently used as a quantitative target during tube repositioning, with real‐time confirmation at the incision site.

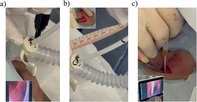

## 1 Introduction

Percutaneous dilatational tracheostomy (PDT) is a bedside technique widely used in patients requiring prolonged mechanical ventilation [1, 2, 3]. The procedure involves puncturing the anterior tracheal wall and inserting a tracheostomy cannula while adjusting the position of an endotracheal tube (ETT). Although complication rates are comparable to surgical tracheostomy [3, 4], ETT‐related events including cuff rupture, guidewire misplacement and inadvertent extubation due to excessive tube withdrawal remain well‐recognized risks [4]. Several approaches have been proposed to optimize ETT positioning, including direct visualization through the glottis [1, 2], bronchoscopic guidance [2] and ultrasonographic cuff identification [5]. However, these approaches may be limited by restricted visualization or procedural complexity and a standardized approach has yet to be established [2, 3]. This report describes a practical adjunct to bronchoscopy‐guided PDT: a two‐step method in which an individualized ETT withdrawal distance is estimated using bronchoscopic transillumination and subsequently used as a quantitative target during tube repositioning, with real‐time confirmation at the incision site.

## 2 Case Report

A 46‐year‐old man with a history of moyamoya disease and vasospastic angina, under outpatient management with oral telmisartan, diltiazem, isosorbide dinitrate and sublingual nitroglycerin, was brought to the emergency department following a sudden loss of consciousness while eating. Written informed consent was obtained for publication of this case report.

Upon arrival, his Glasgow Coma Scale (GCS) score was E1V1M2. Pupillary diameters were 2.0 mm (right) and 3.0 mm (left). Vital signs were: respiratory rate, 18 breaths/min; heart rate, 98 beats/min; oxygen saturation, 98% in room air; and blood pressure, 159/117 mmHg. Neurological examination revealed right conjugate deviation, left hemiparesis, left facial droop and snoring.

Head computed tomography (CT) revealed intraventricular haemorrhage with ventricular rupture. Although hydrocephalus resolved and ventricular drains were removed on hospital day 9, follow‐up CT revealed persistent diffuse ischaemic changes in both cerebral hemispheres with elevated intracranial pressure. Because prolonged mechanical ventilation was anticipated due to persistent impaired consciousness, tracheostomy was scheduled on hospital day 10.

Preoperative laboratory results showed no coagulopathy. Body mass index was 25.1 kg/m^2^ (height, 170 cm; weight, 72.5 kg). At our institution, percutaneous tracheostomy criteria include (a) > 25 mm cricoid cartilage‐sternal notch distance and (b) < 25 mm pretracheal soft‐tissue depth. In this patient, these values were 60 mm and 12.4 mm, respectively; therefore, a percutaneous approach was selected.

**FIGURE 1:**
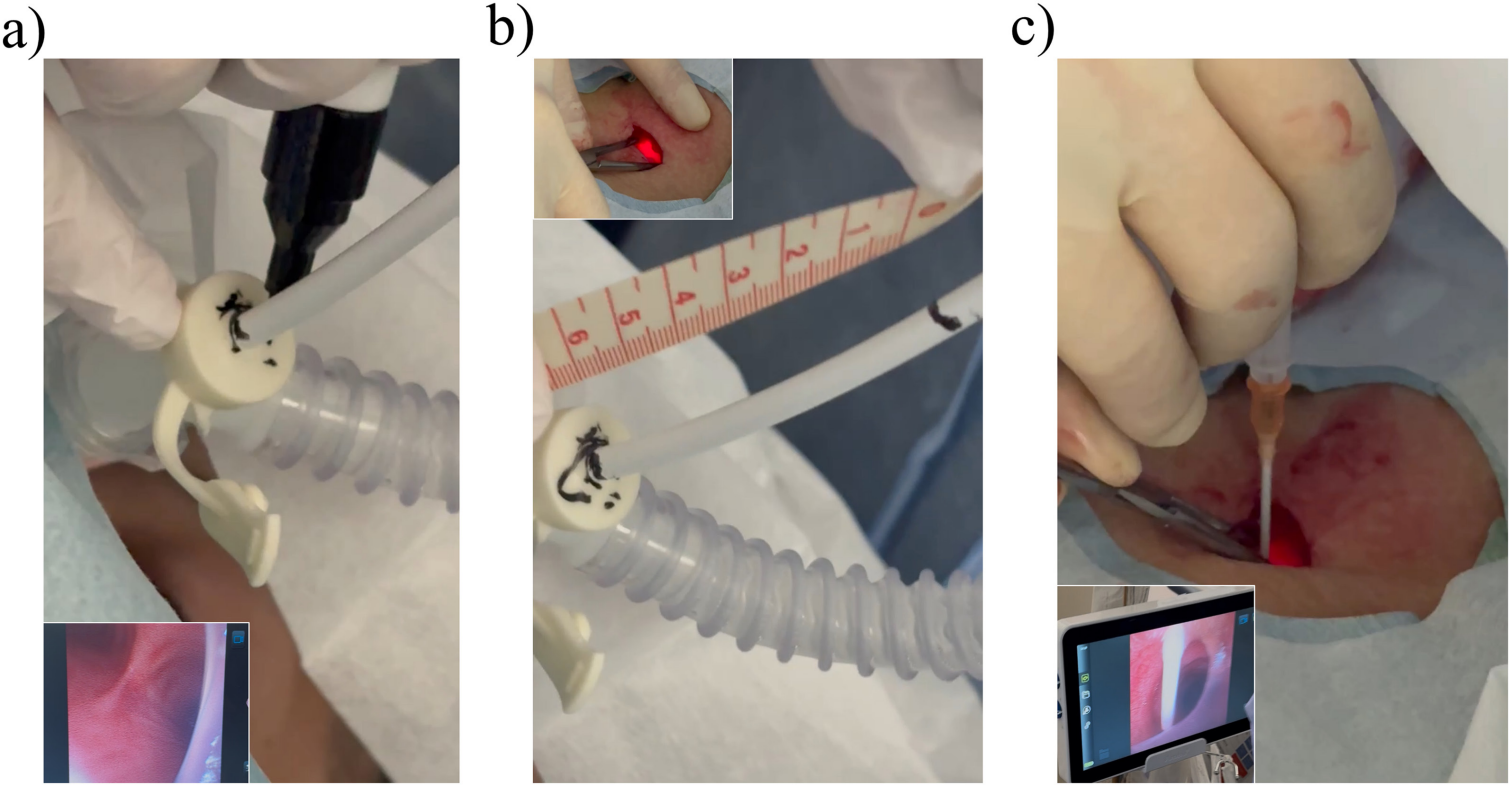
Bronchoscopic light‐guided withdrawal‐distance measurement and tracheal puncture. (a) Alignment and marking of the bronchoscope relative to the ETT. After aligning the distal tips of the ETT and bronchoscope, the bronchoscope was marked at the proximal (oral) opening of the ETT. The bottom left panel shows a bronchoscopic view. (b) Determination of the appropriate ETT withdrawal distance using bronchoscopic light. With the ETT kept in position, the bronchoscope was withdrawn until the light was most clearly visible at the incision site and the bronchoscope was then marked at the proximal (oral side) opening of the ETT. The distance between this mark and the previous mark was measured and defined as the appropriate ETT withdrawal distance. (c) Simultaneous withdrawal of the ETT and bronchoscope by the pre‐measured distance. With the distal tips of the ETT and bronchoscope aligned, both were withdrawn together using the previously measured appropriate length. During withdrawal, bronchoscopic light was visualized from the incision to confirm that it had passed just beneath the incision, indicating correct positioning. The bottom left panel shows a bronchoscopic view. ETT, endotracheal tube.

**FIGURE 2:**
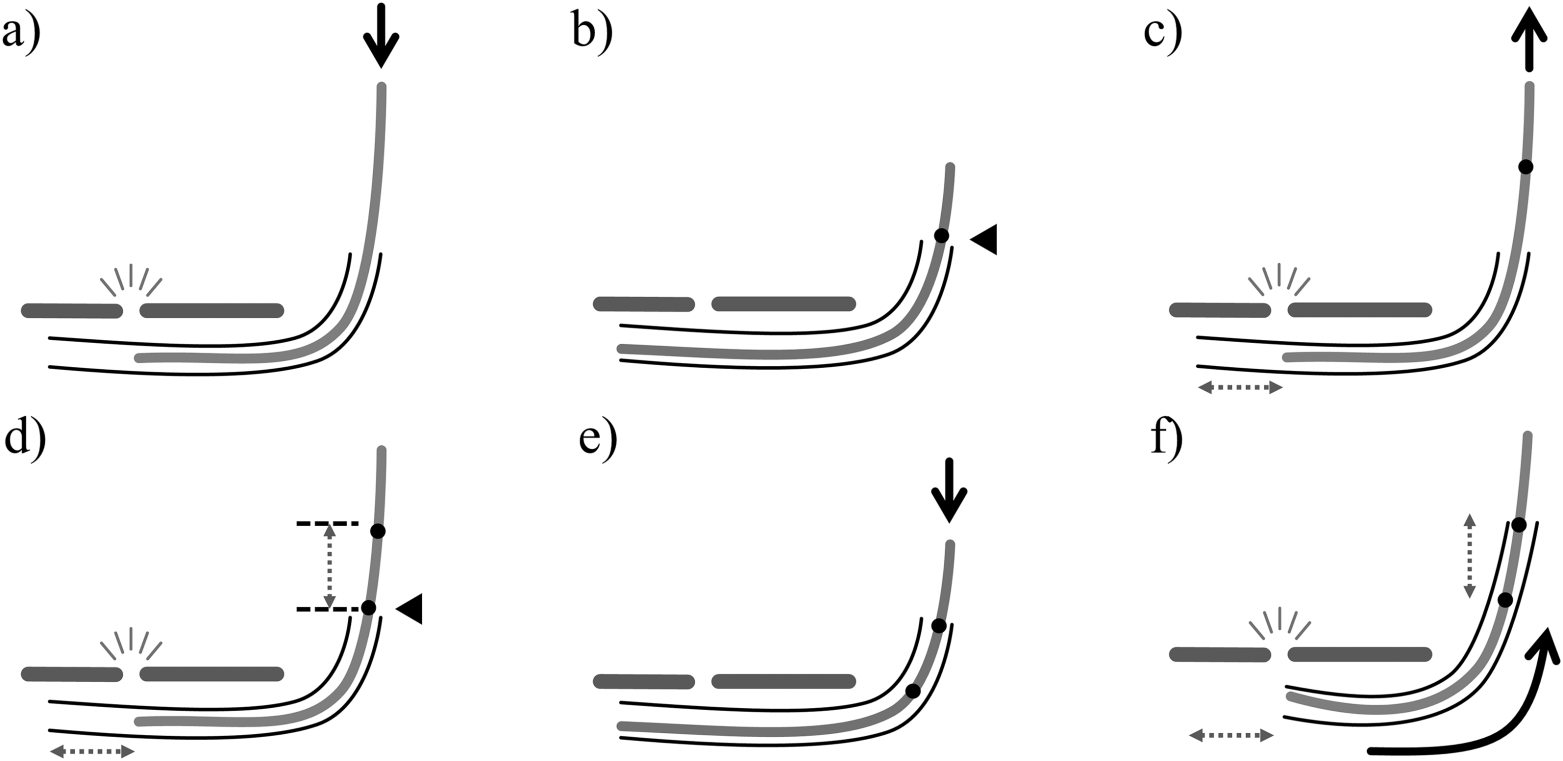
Step‐by‐step schematic of the procedure. (a) Confirmation of bronchoscopic light at the incision site. (b) Bronchoscope marking with ETT and bronchoscope tips aligned. (c) Bronchoscope withdrawal to maximize light at the incision. (d) Measurement of the appropriate withdrawal distance. (e) Re‐alignment of the bronchoscope and ETT tips. (f) Simultaneous withdrawal guided by pre‐measured length and light visualization. ETT, endotracheal tube.

The tracheostomy was performed at the bedside in the intensive care unit. Figure 1 shows the surgical site and bronchoscopic view and Figure 2 presents a schematic illustration of the procedure. A horizontal skin incision (~1.5 cm) was made above the second and third tracheal rings and subcutaneous dissection continued until the tracheal cartilage could be palpated. The ETT and bronchoscope tips were aligned and a reference mark was placed on the bronchoscope (Figures 1a and 2b). The bronchoscope was withdrawn until its light became visible through the incision and passed slightly beyond the puncture site (Figures 1b and 2c), where a second mark was made (Figure 2d). The distance between the two markings defined the ETT withdrawal distance (Figures 1b and 2d). The ETT and bronchoscope were then withdrawn together by the calculated distance. When the bronchoscopic light was visible beneath the incision, ETT withdrawal was halted. Tracheal puncture was performed under bronchoscopic visualization (Figures 1c and 2e), followed by guidewire insertion. The stoma was progressively dilated, and a tracheostomy cannula was inserted. The procedure lasted approximately 20 min with minimal bleeding and no intraoperative complications.

## 3 Discussion

Bronchoscopic guidance is widely used during PDT, and anatomical landmarks such as the tracheal rings or cricoid cartilage can help determine when to stop proximal ETT withdrawal. However, bronchoscopic identification of these landmarks may be challenging when visualization is impaired, potentially leading to variability in ETT repositioning between operators and assistants. The novelty of this technique lies not in bronchoscopy or transillumination itself, but in converting ETT withdrawal into a pre‐measured, quantitative target shared between the proceduralist and the airway assistant.

ETT repositioning during PDT carries risks, including cuff rupture and inadvertent extubation [4]. Although bronchoscopic visualization of the cricoid cartilage can serve as an objective landmark during ETT withdrawal, this landmark may be difficult to identify when visualization is impaired by secretions, airway inflammation or inadequate focus in the proximal airway. Because this landmark is located immediately below the glottis, loss of visualization at this point may further increase the risk of inadvertent extubation. Preventing these complications requires precise coordination and a clear understanding of the intended tube movement. Miscommunication may result in insufficient or excessive withdrawal, thereby compromising safety. To address this issue, this technique involves measuring the optimal ETT withdrawal distance before manipulation, thereby providing an objective reference for accurate tube adjustment.

PDT is typically performed by puncturing between the second and fourth tracheal rings [2]. Before puncture, the ETT tip should remain proximal to the puncture site and distal to the vocal cords to avoid extubation. In this technique, the pre‐measured withdrawal distance is used to guide repositioning, and the ETT position is confirmed by bronchoscopic illumination visible at the incision site. This light‐guided approach may help prevent unintentional extubation caused by excessive withdrawal and may also reduce the risk of cuff or bronchoscope damage due to insufficient withdrawal.

This technique may facilitate more consistent ETT repositioning and requires no specialized equipment beyond conventional bronchoscopic tools. However, its feasibility depends on patient anatomy because it relies on transillumination. In patients with obesity, increased pretracheal soft‐tissue thickness or a long skin‐to‐trachea distance, visualization of the bronchoscopic light may be difficult. Preprocedural assessment of the skin‐to‐trachea distance and vascular mapping may help identify suitable candidates. When transillumination is inadequate or the anatomy is unfavourable, standard bronchoscopic guidance alone or surgical tracheostomy should be considered.

In conclusion, combining pre‐measurement of the ETT withdrawal distance with incision‐site transillumination during PDT may reduce ETT‐related technical difficulties and improve procedural consistency.

## Author Contributions

Y.A. and T.Y. drafted and revised the manuscript. Y.A., T.Y. and M.K. critically revised the manuscript for important intellectual content. All authors read and approved the final manuscript.

## Ethics Statement

The authors have nothing to report.

## Consent

The authors declare that written informed consent was obtained for the publication of this manuscript and accompanying images and attest that the form used to obtain consent from the patients complies with the Journal requirements as outlined in the author guidelines.

## Conflicts of Interest

The authors declare no conflicts of interest.

## Data Availability

The data that support the findings of this study are available from the corresponding author upon reasonable request.
